# Evaluation of a Tasteless Enrofloxacin Pharmaceutical Preparation for Cats. Naive Pooled-Sample Approach to Study Its Pharmacokinetics

**DOI:** 10.3390/ani11082312

**Published:** 2021-08-05

**Authors:** Lilia Gutierrez, Graciela Tapia, Eduardo Gutierrez, Hector Sumano

**Affiliations:** 1 Departamento de Fisiología y Farmacología, Facultad de Medicina Veterinaria y Zootecnia, Universidad Nacional Autónoma de México, Avenida Universidad 3000, Coyoacán 04510, Mexico; liliago@unam.mx (L.G.); dixy1@hotmail.com (E.G.); 2 Departamento de Genética y Bioestadística, Facultad de Medicina Veterinaria y Zootecnia, Universidad Nacional Autónoma de México, Avenida Universidad 3000, Coyoacán 04510, Mexico; tapiadoctora@gmail.com

**Keywords:** enrofloxacin-alginate, pharmacokinetics, cats, naïve pooled sampling

## Abstract

**Simple Summary:**

Enrofloxacin has low oral bioavailability in cats. Additionally, its unpleasant taste is linked to profuse salivation and vomiting, and the cat’s refusal to accept the following dose. In this trial, the pharmacokinetics (PK) of a new pharmaceutical preparation of enrofloxacin-alginate dried beads (DABE) is presented. It eliminates the unpleasant responses of cats to standard oral enrofloxacin. Its PK was carried out under a naive pooled sampling model. PK of DABEs (10 mg/kg/day) concealed in the cat’s food or morsels, complies well with the PK/pharmacodynamics ratios required for most pathogens, i.e., Cmax/CMI > 10. No rejection of DABEs was observed. The retinopathy associated with enrofloxacin appears to be dose-dependent. However, the reported Cmax values observed after the SC administration of 5 mg/kg/day, are notoriously similar to the obtained Cmax value obtained for DABEs (2.3 µg/mL). Hence the higher dose utilized PO is likely to result in similar toxicity as the dose utilized after parenteral administration, with the added benefit of an effortless drug administration and lack of tissue reactions in the injection site.

**Abstract:**

Available pharmaceutical preparations of enrofloxacin injected SC or IM to cats are likely to cause adverse tissue reactions in the injection sites (pH of the drug preparations is ≥10.4). Tablets often induce abundant ptyalism and vomiting, casting doubt on the efficacy of the drug administration maneuver. In addition, the reported oral bioavailability is very low. In this trial, the oral pharmacokinetics of dried alginate beads of enrofloxacin (DABE) administered by concealing them in the cat’s moist food or morsels, is described. A naïve polled sampling approach was chosen with fourteen adult healthy cats. Neither their housing nor their feeding habits were altered. A single pharmacokinetic profile was obtained with 5 samples per designated bleeding time, sampling each cat 2–3 times only. None of the cats rejected their medicated food or morsels. Plasma profile of enrofloxacin exhibited an AUC_0–24_ value of 12.4 µg·h/mL and an AUC_0–∞_ value of 19.2 µg·h/mL, which are comparatively greater than values previously referred for kittens. In contrast, λ and elimination t½ were almost identical (0.12 1/h and 6.1 h). Pharmacokinetics/pharmacodynamics ratios taking the breakpoint of *Staphylococcus epidermidis* as a surrogate (0.5 µg/mL), can be regarded as borderline or low, but perhaps adequate in cats, as higher concentrations may be linked to toxicity (AUC_0–24_/MIC = 24.8; Cmax/MIC = 4.6).

## 1. Introduction

Although enrofloxacin has been on the market for cats for more than three decades and is recommended for various bacterial infections in this species [1,2,3,4], a proper pharmaceutical preparation suitable for them, has not been developed. It is currently approved in the United States by the Food and Drug Administration, as well as in many countries for use in cats. Yet, clinicians are aware of the fact that when regular tablets are administered orally to cats, important undesirable side-effects are often observed, such as exacerbated ptyalism and vomiting. Furthermore, clinicians know that in cats, the initial experience of dosing them with enrofloxacin causes them to refuse a second or third dose of enrofloxacin, making it very difficult to complete a proper dosing scheme. When injected, the procedure has to be repeated every 24 h, and on top of the handling of a non-cooperative cat, local tissue reactions are often observed, making it difficult to comply with dosing schemes, particularly long ones. This may be caused by the considerably high pH of most preparations (approximately 10.4). These peculiarities when administering enrofloxacin to cats necessarily compromise the success of the treatment, and also impose some form of danger to the physical integrity of the owner and/or veterinarian, in their attempt to force oral medication or inject the cat. This is particularly evident when long-term dosing schemes are recommended, such as in the case of feline *Mycoplasma haemofelis* (previously *Haemobartonella felis*) infections that respond to treatment with enrofloxacin at 10 mg/kg/day, for 21 days [5].

The literature often refers to retinopathy, as the most important adverse drug reaction to enrofloxacin in cats [6,7]. Yet, it is not very common, and it is recognized that it is linked to the administration of high doses (≥20 mg/kg/day). Additionally, it has been shown that it is associated with a particular genetic predisposition [8]. In contrast, and despite its common occurrence in practice, not much reference is made in the literature on the already mentioned difficulties associated with prolonged treatment with oral enrofloxacin. An additional consideration that needs to be accounted for is that it has been accepted that higher doses are generally more likely to reach targeted pharmacokinetics/pharmacodynamics (PK/PD) ratios, i.e., maximum serum concentration of enrofloxacin/minimum inhibitory concentration (MIC) of a given pathogen = 10–12; and area under the serum concentration vs time curve in 24 h/MIC = 120 (Cmax/MIC ≥ 10, and AUC_0–24_/MIC ≥ 120, respectively), and achieving these ratios is associated with higher clinical efficacy [9,10]. However, formal pharmacokinetic studies of enrofloxacin in adult cats are scarce. Some plasma concentration data have been presented [3,11], and the only formal pharmacokinetic study was carried out in 6 to 8-week-old kittens. In it, oral administration of enrofloxacin did not result in therapeutic drug concentrations [9].

Recently, it was shown that the pharmaceutical preparation of dried-alginate beads of enrofloxacin (DABE) concealed in a morsel resulted in both adequate pharmacokinetic (PK) parameters and PK/PD ratios in dogs. It appears that these beads are tasteless to dogs, and can be concealed in morsels such as a piece of sausage or pate, without affecting its pharmacokinetics [12]. In the case of cats, it could be useful to define the pharmacokinetics of DABE when included in the moist canned commercial food for cats or morsels. This was the impetus of the present study.

## 2. Materials and Methods

### 2.1. Animals and Sampling

All study procedures and animal care activities were carried out following the Institutional Committee for Research, Care, and Use of Experimental Animals of the National Autonomous University of Mexico (UNAM), under Official Mexican Regulation NOM-062-ZOO-1999. Taking into account the difficulties of working under the SARS-COVID-19 pandemic conditions, and to have an approximation of the variations that may occur when dosing enrofloxacin-alginate beads concealed in the cat’s food when these animals are within their customary environments, this pharmacokinetics study was designed as a naïve pooled sample PK study [13]. This was thought to allow minimum manipulation of healthy household cats. Thus, fourteen young adult cats 2 to 4 years old and weighing from 3.5 to 5.2 kg (mean 4.3 ± 0.66 kg), clinically healthy were included in this trial. They were all fed with canned moist food (Hill’s Liver and Chicken Entrée for adult cats, Mexico City), and they were left within their regular housing. It was ensured that all participant cats did not receive any medication for the last 21 days and a basal sample was obtained in all cats 24 to 48 h before commencing the trial. Then, cats were medicated by adding the necessary number of DABEs in one-fifth of their standard meal (approximately 10–15 g/kg of a moist meal) to allow full consumption of the total dose (10 mg/kg i.e., 12 to 25 DABEs) in the shortest period possible. In some cats, a morsel concealing the DABEs (piece of sausage, piece of fish, or another protein-rich morsel) was allowed. When their complete dose was ingested, it was ensured that no-DABE was left on their plate and then the rest of their food was offered. Then 5, and at some points, 4 blood samples were obtained for each of the following times: 1, 2, 4, 8, 12, and 24 h. Excepting cats number 1 and 7 who were samples thrice in 24 h, no cat was bled more than twice in that time (see Table 1), and blood was obtained with heparin primed Vacutainer tubes and venipuncture pediatric butterfly needles. The subjects were selected randomly, and blood samples from each subject were taken in a way that they covered the entire range of concentrations versus time pre-determined data profile.

### 2.2. Manufacturing DABE

**Figure 1:**
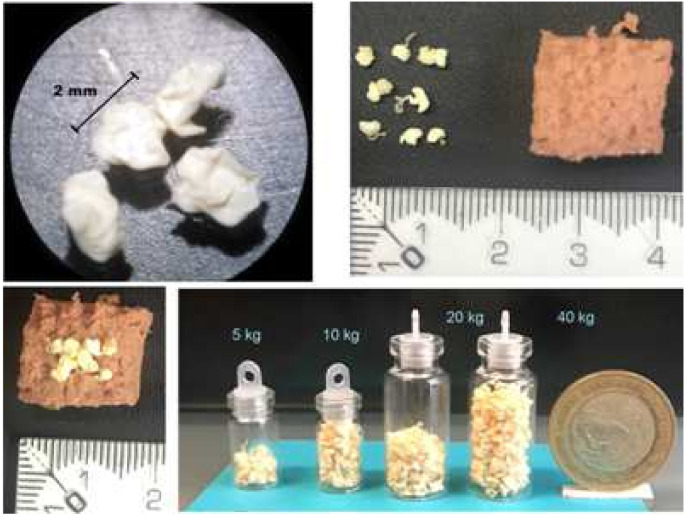
Aspects of the shape and size of dried alginate-beads of enrofloxacin (DABE) and how to conceal them in cat’s food.

Alginate-dried beads of enrofloxacin were manufactured as previously described [12]. Thus, 2.5 mg of enrofloxacin are contained in each bead and cats were dosed with 10 mg/kg of enrofloxacin; hence, a 4 kg cat will need 16 beads per day in a single dose. The DABE were obtained as yellowish-color particles with a relatively smooth surface and irregular confetti or drop-like morphology with an average size of 1.52 ± 1.0 mm. Figure 1 shows DABEs and how DABEs were concealed in the cat’s food.

### 2.3. Quantification of Plasma Concentrations of Enrofloxacin

The method of enrofloxacin quantification in plasma samples was developed and validated in our laboratory and was based on the method described by Idowu and Peggins [14]. An aliquot (1 mL) of plasma was added to 1 mL of methylene chloride and, after shaking the mixture for no more than 10 s on a vortex mixer, it was centrifuged for 5 min at 11,200× *g*. The supernatant was discarded and the organic phase was evaporated in a nitrogen environment. Residues were reconstituted in acetonitrile:methanol:water (17:3:80), with phosphoric acid (4% *v*/*v*), and trimethylamine (4% *v*/*v*) as the mobile phase. This product was then analyzed by high-performance liquid chromatography (HPLC) using a Hewlett Packard 1046A fluorescence detector at *λ*_exc_ 280 nm and *λ*_em_ 460 nm. A Jasco XLC HPLC system (LC-2000Plus; Jasco Benelux, Utrecht, The Netherlands) with a Symmetry-C18 column (4.6 mm × 100 mm, 3.5 μm; Waters, Milford, MA, USA) was used. Injection volume was 50 μL and flow was 0.6 mL/min. Data were analyzed by using Empower 3 from Waters (Mexico City, Mexico). The chromatographic method was validated, and the analytical procedure was demonstrated as specific. The method produced a linear result from 0.01 to 20.48 μg/mL (*r*^2^ = 0.984; *y* = 500,030; *x =* 107,046). Recovery of enrofloxacin was calculated by applying linear regression analysis. Samples had an *r*^2^ = 0.978 (*y* = 0.072322309; *x* = 0.1233375). Precision was demonstrated by the inter-day coefficient of variance (3.1) and inter-assay error value (<3.9). The lower quantification limit for enrofloxacin in plasma was 0.01 μg/mL with a detection limit of 0.008 μg/mL, and linearity was established up to 10 μg/mL. For robustness and tolerance, an absolute difference of 1.8 and a coefficient of variance of 2.2% (<3.0%) were obtained.

### 2.4. Pharmacokinetics

The concentrations versus time data were analyzed both by compartmental and noncompartmental analysis (PKAnalyst; MicoMath, Salt Lake City, USA and WinNonlin version 5.2.1; Pharsight Corporation, Mountain View, CA, USA respectively), assuming that the plasma concentrations of enrofloxacin vs time data obtained were from a single subject. Results presented include Cmax (maximum plasma concentration); Tmax (time to reach Cmax); T½_λ_ (elimination half-life); AUC_0–24_ (area under the plasma concentrations versus time curve in 24 h); AUC_0–∞_ (area under the plasma concentrations versus time curve from 0 to ∞); AUMC_0–∞_ (area under the moment curve from 0 to ∞) and MRT (mean residence time).

### 2.5. Monte Carlo Simulation

Monte Carlo simulation was used to generate the probabilities of target attainment (PTA) of both accepted PK-PD ratios for enrofloxacin (C_max_/MIC ≥ 10, and AUC_0–24_/MIC ≥ 125) at a level of ≈90% or higher, and using 1000 sham cats (WinNonlin version 5.2.1; Pharsight Corporation). The choice of the bacterial sensitivity to enrofloxacin was adopted arbitrarily from the available literature [1,10,15].

## 3. Results

**Figure 2:**
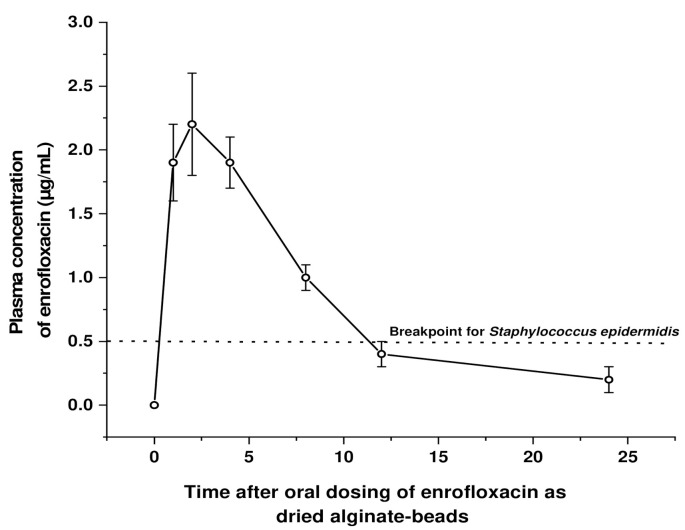
Mean ± 1 SD plasma concentrations of enrofloxacin after a single dose of the drug prepared as dried alginate beads of enrofloxacin (DABE). Fourteen healthy adult cats enter the study and samples were obtained through a naive pooled sampling model, having 4 to 5 determinations of enrofloxacin per time. For visual reference, the breakpoint for *Staphylococcus epidermidis* has been incorporated.

All participant cats received a 10 mg/kg dose of enrofloxacin as DABE, concealed in their food or a morsel, and none of them rejected their medicated food or exhibited behavior that could indicate that the food had changed in taste. Table 2 and Figure 2 present values of the PK parameters obtained for enrofloxacin by the non-compartmental and compartmental analysis, and the plasma concentrations vs time profile obtained, and Table 1 shows the sampling arrangement of the 14 healthy adult cats included in this trial to draw 2–3 blood samples from each cat to end with an η of 5 per sampling time and the whole set for the basal samples at time zero. Additionally, taking the breakpoint of *Staphylococcus intermedius* as a surrogate value (0.5 µg/mL) [1,10,15,16], pharmacokinetics/pharmacodynamics (PK/PD) ratios are presented. Table 2 also presents, as a comparison for this naïve pooled sampling PK data, the mean ± SD of the available data on the pharmacokinetics of orally administered enrofloxacin in 6 to 8 weeks old kittens [9].

**Figure 3:**
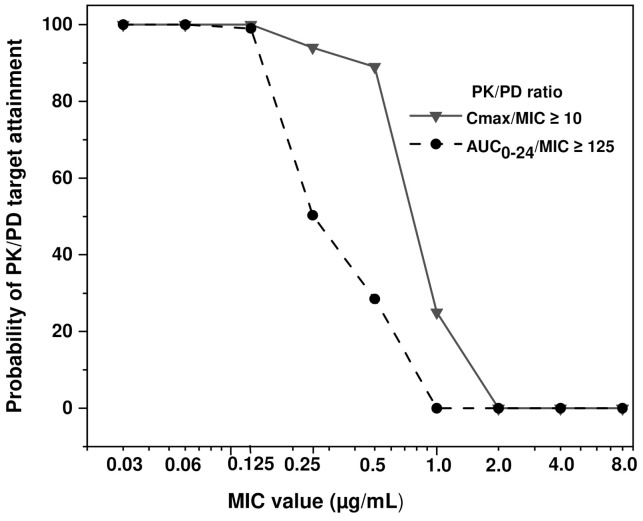
Monte Carlo simulations for dried alginate beads of enrofloxacin (DABE), administered to cats at doses of approximately 10 mg/kg and concealed in their food. Potentially pathogenic bacteria with sensitivities that range from 0.03 to 8 ug/mL were considered, and both known PK/PD ratios for enrofloxacin were incorporated.

As far as Monte Carlo simulations are concerned, the percentage of sham patients who achieved Cmax/MIC ≥ 10, and AUC_0–24_/MIC ≥ 125 ratios in each MIC value are presented in Figure 3. For the AUC_0–24_/MIC ≥ 125 ratio, it was found that PTA was 90–100% with MICs from 0.03 to 0.5 µg/mL. When MIC value is 1 µg/mL, only 65% PK/PD adequacy was predicted, and 10% for a MIC value of 2.0 µg/mL. For the Cmax/MIC > 10 ratio the PTA was 100% for MIC values of 0.03 to 0.125; 94% for 0.25 µg/mL, and 89% for 0.5 µg/mL. Hence, the PK/PD cut-off point was approximately 0.5 µg/mL, considering a dose of 10 mg/kg of enrofloxacin as DABEs. 

## 4. Discussion

A review of the main databases to search for PK studies of enrofloxacin in cats revealed only one study in this species. It was carried out in kittens and authors found that after oral administration of the drug, bioavailability was so low that the serum concentrations obtained, were negligible [9,18]. The only other study that has been carried out to define the PK of enrofloxacin in cats only evaluated IV and SC administration of the chemical derivative enrofloxacin-HCl [17]. In this latter study, oral-PK was not done and some discrepancies can be highlighted when compared to the former. For example, a longer half-life in the former study was found (T½ elimination = from 4.6 to 7.3 h), as compared to data obtained by the latter authors (T½β = 3.9 to 4.6 h). No other studies were found that formally evaluated the oral pharmacokinetics of enrofloxacin in cats. Yet, other authors presented the concentrations achieved with the subcutaneous injection of 5 mg/kg enrofloxacin, but without calculating PK parameters from the observed plasma profile of the drug [3].

In this trial, useful serum concentrations were found after dosing the cats with 10 mg/kg of enrofloxacin-alginate dried beads (DABEs), which were easily mixed/administered in the cats’ food or morsel. Cmax values in this trial were 2.3 µg/mL vs. 0.8 µg/mL found in kittens [9]. This study was developed under a naïve pooled sampling model due to various considerations such as the trial was carried out during the confinement period imposed during the COVID-19 pandemic in the world, but also due to the fear of the owners of generating retinopathy in their pets, and because we, the authors, could not conciliate the idea of acquiring experimental cats and subjecting them to the stress of more than 2 blood-samplings per day, while keeping them in cages. The proposed advantages of this experimental design are that a descriptive structural model is fit to data from all individuals as if they were one individual and PK data can be expanded to a population PK. Of course, making assumptions regarding the form of the variation in each parameter between individuals and regarding the distribution of measurement errors, was necessary. The individual’s concentration profile is assumed to be similar to the “population” profile [19,20].

For susceptible isolates, enrofloxacin consistently reached PK/PD indices associated with clinical efficacy, but in this case, a relatively high dose was utilized i.e., 10 mg/kg. Boothe et al. [10] presented MIC mean values for Gram-negative bacteria of 0.17 µg/mL for *Proteus mirabilis* to 0.45 µg/mL for *Pseudomonas aeruginosa*, and for Gram-positive pathogens from 0.84 µg/mL (*Staphylococcus* spp.) to 0.19 µg/mL for *Staphylococcus intermedius*. These MIC values fall into the ranges utilized for the Monte Carlo simulations carried out in this study. Enrofloxacin is lipid-soluble and a zwitterion, and as such, it is reasonable to think that it has good distribution to many tissues, as it happens with other species [2,21]. This has been shown for cats receiving a low dose of 5 mg/kg of enrofloxacin IM, and with this dose, therapeutic concentrations of the drug were found in tears, saliva, and serum of healthy cats or cats with signs of upper respiratory tract infection. In this trial, no side effects were observed. However, it has been commented after in vitro PK/PD studies that it is important to achieve high C_max_ values to mitigate the selection of new fluoroquinolone-resistant strains and consistently reach PK/PD indices associated with adequate clinical efficacy. This occurs only at the highest dose possible [10,22,23]. Hence, the chosen dose of 10 mg/kg for this study.

The incidence of retinal degeneration and blindness in cats medicated with enrofloxacin occurs sporadically in cats with a proposed rate of 0.008% [23] and is most commonly observed when administering high parenteral doses of enrofloxacin; for example, higher than 5 mg/kg/d IV or SC [24,25]. This adverse reaction is more likely to occur at a dose of ≥20 mg of enrofloxacin/kg/d in adult cats and again, administered preferably after IM, or SC injection, given bioavailability values >85% through these routes [9]. In contrast, this adverse event is less likely to occur with a dosage of 5–10 mg of enrofloxacin/kg/d administered orally. This may be due to the low oral bioavailability of enrofloxacin, which one of the few studies available has revealed [9]. In this trial, no formal examination of the retina was attempted to rule out damage by enrofloxacin. However, it was assumed that retinal damage did not occur given the absence of typical clinical signs of vision impairment in cats such as disorientation and bumping into things, walking cautiously, and unwillingness to jump, tendency to hide away, and becoming nervous or any other change in their behavior. As with many other drugs, the potential benefits of administering enrofloxacin should be outweighed by this and other side effects. Nevertheless, further evaluation is necessary when using the enrofloxacin-alginate pharmaceutical preparation for oral administration, here studied. This is particularly relevant because it is well documented that the parenteral administration in cats at doses greater than 5 mg/kg should not be considered because of reported retinal toxicity, and because the parenteral administration of standard enrofloxacin solutions, can cause severe pain and tissue damage [24].

To obtain more regular PK profiles of enrofloxacin in cats the drug should be administered orally on an empty stomach as food is known to affect the absorption of fluoroquinolones [4,26]. However, the temperament of the cat makes this task difficult, and resort to IM or SC administration of enrofloxacin entails many side effects both in the injection site, and potentially making retinal toxicity more likely to occur [6,23,24] Hence, despite the approval in many parts of the world of parenteral administration of enrofloxacin, this route should be reserved for those cases in which bacterial susceptibility has been demonstrated [10,21,27]. In this context, enrofloxacin-alginate at 10 mg/kg achieves reasonably good PK/PD ratios [28,29], and it is easily and uneventfully administered to cats in their food, and DABEs can also be offered to cats, concealed in their preferred morsels. Finally, given the risk of retinal toxicity induced by enrofloxacin in cats, further studies are necessary to adjust DABEs dosage in cats with renal insufficiency [30].

## 5. Conclusions

Standard enrofloxacin has low oral bioavailability in cats; additionally, it has a bitter-unpleasant taste that induces a notorious ptyalism and often vomiting. The new pharmaceutical preparation of enrofloxacin-alginate dried beads (DABE) showed that cats presented no reaction when administered concealed in their food or within a morsel. Its PK through naive pooled sampling and Monte Carlo simulations shows that DABEs administered at 10 mg/kg/day comply well with the main PK/PD ratios required for most pathogens, and C_max_ after dosing 10 mg/kg achieves a similar C_max_ as the reported value of this parameter, after the SC injection of enrofloxacin (5 mg/kg). Finally, as with many other drugs, the potential benefits of administering enrofloxacin as DABEs should be pondered against the known side effect of this active principle, but it must be considered that the described key PK parameters of DABEs at 10 mg/kg/day are almost identical to the ones obtained after the SC or IM administration of standard solutions of enrofloxacin but without the inflammatory reactions in the injection sites.

## Figures and Tables

**Table 1:** Concentrations of enrofloxacin in the 14 healthy adult cats included in this trial. Two and in some cats three blood samples were drawn under a random design, obtaining 3 mL per cat. In the end, each sampling time had an η of 5, excepting the basal sample which was obtained from all participants.

Sampling Time (h)	Cat Number														$\bar{x}$	±1 SD
	1	2	3	4	5	6	7	8	9	10	11	12	13	14		
zero	0	0	0	0	0	0	0	0	0	0	0	0	0	0	0	0
1	NS	NS	NS	1.6	NS	2	2.3	NS	NS	NS	NS	1.8	1.8	NS	1.9	0.3
2	1.8	NS	NS	NS	NS	NS	NS	NS	1.9	2.6	2.4	NS	NS	2.4	2.2	0.4
4	NS	2	1.8	NS	2.2	NS	1.6	1.8	NS	NS	NS	NS	NS	NS	1.9	0.2
8	0.9	NS	NS	NS	NS	1.1	NS	0.9	NS	NS	NS	1.2	NS	0.9	1	0.1
12	NS	0.5	NS	NS	0.4	NS	0.4	NS	NS	0.3	NS	NS	0.4	NS	0.4	0.1
24	0.08	NS	0.1	0.2	NS	NS	NS	NS	0.3	NS	0.4	NS	NS	NS	0.2	0.1

NS = the cat was not sampled.

**Table 2:** Pharmacokinetics (PK) parameters for enrofloxacin prepared as dried alginate beads of enrofloxacin (DABE) in 14 healthy adult cats whose blood was obtained through a naive pooled-sampled model, having 4 to 5 determinations of enrofloxacin per time. In comparison, the mean PK values of enrofloxacin after the administration of a tablet, reported in 6 to 8-week-old kittens, is presented [9].

Parameter	Enrofloxacin	
	From DABE	As Tablet Data from [9]
AUC_0–∞_ (µg·h)/mL)	19.2	7.1 ± 3.7
AUC_0–24_ (µg·h)/mL)	12.4	NA
*λ* (1/h)	0.12	0.15 ± 0.07
Elimination t½ (h)	6.1	5.7 ± 2.1
C_max_ (µg/mL)	2.3	0.8 ± 0.36
T_max_ (h)	2.2	NA
MRT (h)	12.5	NA
PK/PD ratios		
AUC_0–24_/MIC *	24.8	NA
C_max_/MIC *	4.6	NA

η = 14 cats and 5 samples per bleeding-time. C_max_ = maximum plasma concentration; T_max =_ time to reach C_max_; λ = (elimination rate constant); Elimination t½ = elimination half-life; AUC_0–24 =_ area under the plasma concentrations versus time curve in 24 h; AUC_0–∞_ = area under the plasma concentrations versus time curve from 0 to ∞; and MRT = mean residence time. * MIC/breakpoint for *Staphylococcus epidermidis* = 0.5 µg/mL [17]. NA = not available.

## Data Availability

All datasets generated for this study are available upon request.
